# Non-destructive 3D Microtomography of Cerebral Angioarchitecture Changes Following Ischemic Stroke in Rats Using Synchrotron Radiation

**DOI:** 10.3389/fnana.2019.00005

**Published:** 2019-01-31

**Authors:** Yonghong Luo, Xianzhen Yin, Shupeng Shi, Xiaolei Ren, Haoran Zhang, Zhuolu Wang, Yong Cao, Mimi Tang, Bo Xiao, Mengqi Zhang

**Affiliations:** ^1^Department of Neurology, Xiangya Hospital, Central South University, Changsha, China; ^2^Center for Drug Delivery System, Shanghai Institute of Materia Medica, Chinese Academy of Sciences, Shanghai, China; ^3^Department of Orthopaedics, Second Xiangya Hospital, Central South University, Changsha, China; ^4^Department of Breast Surgery, Hunan Provincial Maternal and Child Health Care Hospital, Changsha, China; ^5^Department of General Surgery, Xiangya Hospital, Central South University, Changsha, China; ^6^Department of Spine Surgery, Xiangya Hospital, Central South University, Changsha, China; ^7^Department of Pharmacy, Xiangya Hospital, Central South University, Changsha, China; ^8^Institute of Hospital Pharmacy, Xiangya Hospital, Central South University, Changsha, China

**Keywords:** ischemic stroke, synchrotron radiation, 3D, vessel remodeling, microtomography

## Abstract

A better understanding of functional changes in the cerebral microvasculature following ischemic injury is essential to elucidate the pathogenesis of stroke. Up to now, the simultaneous depiction and stereological analysis of 3D micro-architectural changes of brain vasculature with network disorders remains a technical challenge. We aimed to explore the three dimensional (3D) microstructural changes of microvasculature in the rat brain on 4, 6 hours, 3 and 18 days post-ischemia using synchrotron radiation micro-computed tomography (SRμCT) with a per pixel size of 5.2 μm. The plasticity of angioarchitecture was distinctly visualized. Quantitative assessments of time-related trends after focal ischemia, including number of branches, number of nodes, and frequency distribution of vessel diameter, reached a peak at 6 h and significantly decreased at 3 days and initiated to form cavities. The detected pathological changes were also proven by histological tests. We depicted a novel methodology for the 3D analysis of vascular repair in ischemic injury, both qualitatively and quantitatively. Cerebral angioarchitecture sustained 3D remodeling and modification during the healing process. The results might provide a deeper insight into the compensatory mechanisms of microvasculature after injury, suggesting that SRμCT is able to provide a potential new platform for deepening imaging pathological changes in complicated angioarchitecture and evaluating potential therapeutic targets for stroke.

## Introduction

Ischemic stroke is currently the leading cause of severe disability and mortality, which shows a tendency of attack among the younger population (Meschia et al., 2014). Considering the tremendous burden associated with stroke, the importance of understanding the pathogenesis to help in the search for more effective treatments cannot be ignored. The microvasculature in the central nervous system shows a delicate and complex three dimensional (3D) structure. Neural tissue survives under normal physiological conditions since the brain microvasculature can provide the necessary nutrition and maintain a balanced local microenvironment (Ergul et al., 2012). Under the pathological process of brain ischemia, acute stroke will severely injure the cerebral vasculature and lead to irreversible damage to the neurological function (Wittko-Schneider et al., 2014). Previous research has revealed that revascularization following ischemic stroke is a complicated process that refers to the remodeling of the connectivity, number, direction, and spatial distribution of blood vessels, all of which influence the functional recovery of the brain from ischemic injury (Font et al., 2010; Ergul et al., 2012). The newly generated vasuculature supports direct nutrients to focal lesions, which is a prerequisite for the survival of neurons and might play a crucial role for recovery and prognosis. However, the complex morphological remodeling of the vascular network involved in the pathological process post-ischemia still remains largely unknown.

In particular, knowledge of the 3D pathologic changes in the brain microvasculature after stroke may be beneficial to the development of meaningful therapeutic strategies to improve its unsatisfactory outcomes (Li et al., 2010; Zhang et al., 2015). At present, imaging techniques for quantitative evaluation of the 3D morphologic changes in the spinal microvasculature in the ischemic process are short of effectiveness. These techniques are most commonly used in diagnosing the vascular pathology through magnetic resonance angiography (MRA), and computed tomography angiography (CTA) in clinical practice (Dorr et al., 2007). However, they have limited competence to provide detailed readouts in small animals. MRA is limited by potential flow artifacts and long acquisition times. Moreover, it is inappropriate to evaluate acute vascular changes. CTA has insufficient resolution to detect microvessels on micrometer or submicrometer scales. Histological sectioning is widely known as the gold standard for characterizing vessel morphology down to the micrometer scale (Zhang et al., 2014b). However, the 3D structural integrity of the brain specimen is destroyed by the sectioning and the vasculature is only provided in two-dimensional (2D) images, while the 3D information is much more unobtainable. Furthermore, the subsequent preparation process including the staining step is usually time-consuming (Heinzer et al., 2006; Cao et al., 2017). Therefore, further development of novel microimaging methods is necessary for non-invasive high-resolution imaging that can detect changes in intricate microvascular networks under physiological and pathological conditions. The recent emergence of a state-of-the-art synchrotron radiation (SR) device has provided coherent and brilliant radiation that is adequate for high-resolution microtomography of thick samples, usurping the diffraction limitation of conventional optical imaging techniques (Shirai et al., 2013; Chen et al., 2014). Notably, SR-based phase contrast imaging (SR-PCI) is particularly suitable for 3D visualization of high image contrast fine microstructures in biological tissues that have weak absorption of x-ray with no addition of contrast agents (Westneat et al., 2008; Gureyev et al., 2009; Bravin et al., 2013).

In the present study, we employed SRμCT to simultaneously detect the temporal and spatial changes in the 3D architecture of cerebral microvasculature induced by acute stroke in a permanent middle cerebral artery occlusion (MCAO) model. Specifically, the dynamic changes of a spatial vascular network in ischemic brain regions are revealed by the reconstructed multi-angle 3D digital mapping. Furthermore, we attempted to show the gradually formed cavity with separate marked colors. Additionally, we acquired plentiful 3D quantitative information about vascular content by virtue of analysis of global network skeleton maps. This study presents novel findings in systematically characterization and quantitative analysis of 3D morphological changes in cerebral angioarchitecture changes following ischemic stroke on a global-local scale. The current analysis will provide vital insight into our understanding of vascular mechanisms underlying the course of brain ischemia and will aid in the development of new therapeutic strategies, thus targeting microvessels in neurovascular diseases.

## Materials and Methods

### Animals

Animal use and care was performed in accordance with the Central South University Ethics Review of Experimental Animal Welfare, Administration Committee of Affairs Concerning Experimental Animals in Hunan Province, China. All experimental protocols were confirmed by the Animal Ethics Committee of Central South University, Changsha, China (4th March, 2015/No. 201503075). A total of 30 Sprague–Dawley® (SD) male rats (250–280 g) were obtained from the Animal Center of Central South University, Changsha, China and randomly divided into five groups (6 rats per group): a sham control group, a group analyzed 4 h after MCAO, a group analyzed 6 h after MCAO, a group analyzed 3 days after MCAO, and a group analyzed 18 days after MCAO. Animals were raised in a temperature-controlled room with a 12-h light/dark cycle and free access to water and food and acclimatized for at least 7 days before use.

### Construction of an Experimental MCAO Model

Adult SD rats were put in a supine position on a pad and were anesthetized with an intraperitoneal (i.p.) injection of 10% chloral hydrate (0.4 ml/kg). Body temperature was kept at 36.5°-37.0°C throughout the surgical procedure using a heat lamp. After the right common carotid artery was isolated, which included internal and external carotid arteries (ICA), a 4–0 nylon suture coated with silicone was inserted through the ICA to the ostium of the MCA. The mean length of the suture inserted was 18 ± 0.5 mm. The effects of occlusion were evaluated using a neurobehavioral experiment described by Longa et al (Longa et al., 1989) immediately following surgery until the rats awoke from anesthesia following surgery. A group of six normal rats that only underwent the same surgical procedure without insertion of the suture served as a sham control group. All experimental animals were raised in acrylic box cages with free access to water and food. They were also kept under a constant temperature (25°C), humidity (50 ± 10%), and lighting cycle (12:12 h).

### Sample Preparation

All animals were deeply anesthetized with 10% chloral hydrate (0.4 ml/kg) ahead of transcardiac perfusion. Paraformaldehyde (4%) in 0.1M phosphate buffer (pH 7.4) was subsequently perfused at different time points for tissue fixation. All the procedure was conducted in a laboratory at 22°C, and all the perfused solutions were preheated to 37°C. Rat brains were then extracted and post-fixed with 4% paraformaldehyde at 4°C for 24 h in PBS. Then they were separated for different experiments. While every 3 rats of every group were observed histologically, the remaining 15 rats were analyzed by SRμCT. Each of the rinsed brain specimens was then dehydrated for 24 h with a gradient of ethyl alcohol and then prepared in a fully dehydrated state for SRμCT scanning (Zhang et al., 2015).

### SRμCT Imaging

Three animals from each group were used for this experiment. Brain samples were immobile at the middle of the rotation stage, which had a height of nearly 10 mm and a width of about 9 mm, and analyzed by SRμCT at the Shanghai Synchrotron Radiation Facility. Images were captured at the Shanghai Synchrotron Radiation Facility BL13W1 Beamline. X-rays were generated from an electron storage ring with an average beam current of 200 mA and an accelerated energy of 3.5 GeV. The beamline included a tunable energy spectrum ranging from 8 to 72.5 keV. The synchrotron radiation beam was monochromatized by a double-crystal monochromator. The sample was fixed on the rotary stage, which was exposed to the SR light path. Subsequently, the SR light transmitted through the object was detected by the charge coupled device (CCD) camera with 5.2 × 5.2 μ m/pixels (Photonic Science, UK). To obtain the fine image contrast, the monochromatic X-ray energy was adjusted to 20 keV, the exposure time was set to 1.5 s, and the sample-to-detector distance (SDD) was adjusted to 50 cm. While the sample stage rotated 180°, a total of 900 initial projecting images were collected (Zhang et al., 2015). In addition, flat-field and dark-field images were also captured during each acquisition procedure to correct the electronic noise and variations in the X-ray source brightness.

### Image Processing and 3D Vascular Quantitative Analysis

Tomographic reconstructions were reconstructed using phase-sensitive X-ray image processing and tomography reconstruction (PITRE) software applied by the BL13W1 experimental station of the SSRF (Chen et al., 2011). A sequential series of 2D slices reconstructed the 3D rendered images by using Image Pro Analyzer 3D (Version 7.0, Media Cybernetics, Inc., USA). The vasculature was extracted from the parenchyma based on the iterative gray level-based threshold algorithm. The input vasculature for centerline extraction was skeletonized with a dendrite analysis method on the basis of morphological algorithms as described previously (Zhang et al., 2015). The procedure mainly included segmentation, skeletonization, and vectorization. The data were binarized setting a threshold for vessel segmentation based on the feature-based algorithm known as “Frangi filter” (Frangi et al., 1998; Sato et al., 1998; Lang et al., 2012). There is a trade-off between capturing blood vessels and avoiding detecting structures induced by noise. It was selected manually by optimal visual inspection (Lang et al., 2012). The centerlines of the vessels were extracted using a skeletonization method by thinning of the binary input vasculature, which provided a direct distance map. Here, the quantification of parameters such as branches, nodes, length, diameter, and global statistics were calculated. All of the 3D quantitative analysis was performed with the Image Pro Analyser 3D software.

### Immunohistochemistry

At the time of euthanasia, the remainder of the animals were perfused as described above. The samples for immunohistochemistry were then cryoprotected with 30% sucrose/PBS overnight, embedded in an optimal cutting temperature compound (OCT) (Sakura Finetek USA) and cut into 10 mm-thick sections. Coronal sections were collected and washed three times with PBS. Antigen unmasking was performed for CD31 using a PBS solution with 1 μg/mL proteinase K (15 min at room temperature). Sections were blocked using PBS containing 0.25% triton and 20% normal Horse Serum, for 90 min at room temperature. Slices were then incubated overnight at 4°C with primary antibody (PBS containing 0.1% triton) against CD31 (1:100; Abcam plc, Cambridge, UK). Sections were washed three times with PBS and incubated with horseradish peroxidase-conjugated secondary antibody (1:200 dilution; Santa Cruz Biotechnology, Santa Cruz, CA, USA) at room temperature for 1 h. Slides were first stained using the Diaminobenzidine (DAB) kit (Maixin, Fuzhou, China) according to the manufacturer instructions and then counterstained with Harris hematoxylin. All fields were photographed using an Olympus BX51 microscope (Olympus, Tokyo, Japan) and compared with the vascular images obtained by using SR imaging.

### Statistical Analysis

Data are presented as the mean ± standard deviation (SD). Data analysis was performed with SPSS 17.0 (SPSS, Inc., Chicago, IL, USA). The data were analyzed with one-way analysis of variance (ANOVA) followed by Tukey's method for the *post hoc* test to observe the effect of time post-ischemia on the morphological parameters. *p*-value < 0.05 was considered to be significant. Raw data as well as ANOVA results are available in the Supplementary Data 1.

## Results

### 3D Surface Volume Rendering Images of Brain After MCAO

**Figure 1:**
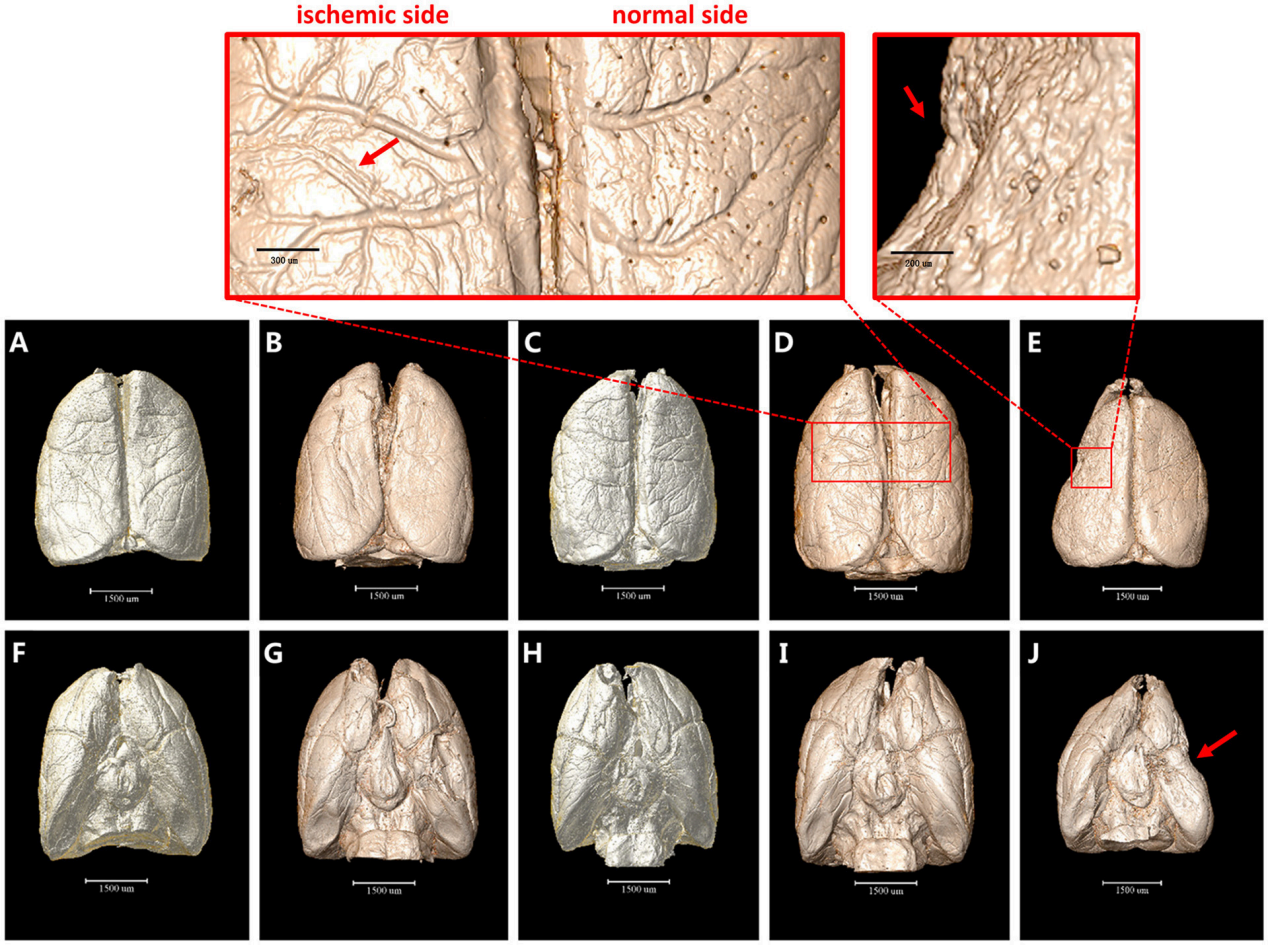
3D surface volume rendering images of brain after MCAO. **(A–E)** top view. **(F–J)** bottom view. **(A)** and **(F)** Sham operation group. **(B,G)** 4 h after MCAO. **(C,H)** 6 h after MCAO. Leptomeningeal vasculatures at 4 h and 6 h after ischemia did not exhibit obvious changes compared to sham operation group. **(D,I)** 3 d after MCAO. Ischemic side began to lose their integrity and organization with small vessels looking stiff and distorted as directed by red arrow in the magnified panel. **(E,J)** 18 d after MCAO. Ischemic side atrophied (red arrow) and collapsed and vascular network decreased. Scale bars: 1,500 μm **(A–J)**, 300 μm (top left) and 200 μm (top right).

The 3D surface volume rendering images showed the changes of leptomeningeal vasculature at 4 h/6 h/3 d/18 d after MCAO (Figure 1). Compared with the sham operation group, leptomeningeal vasculatures at 4 h and 6 h after ischemia did not exhibit obvious changes (Figures 1A–C,F–H). However, 3 d after ischemia, the vascular network displayed in an asymmetric manner with vessels of the normal side being smooth in shape and natural in course while vessels of the ischemic side lost their integrity and organization. Where the red arrow points in the magnified panels, 3 d after MCAO, some small vessels looked stiff and took the shape of withered arborization, and went in a disordered and distorted pattern. Blind endings were formed and rarely but truly, the anastomosed vascular network could be seen (Figures 1D,I). On the 18 d after ischemia, brain of the ischemic side atrophied (red arrow) and collapsed, being smaller in size. The vascular network was not obvious (Figures 1E,J).

### 3D Tomography of Cerebral Angioarchitecture Following MCAO

**Figure 2:**
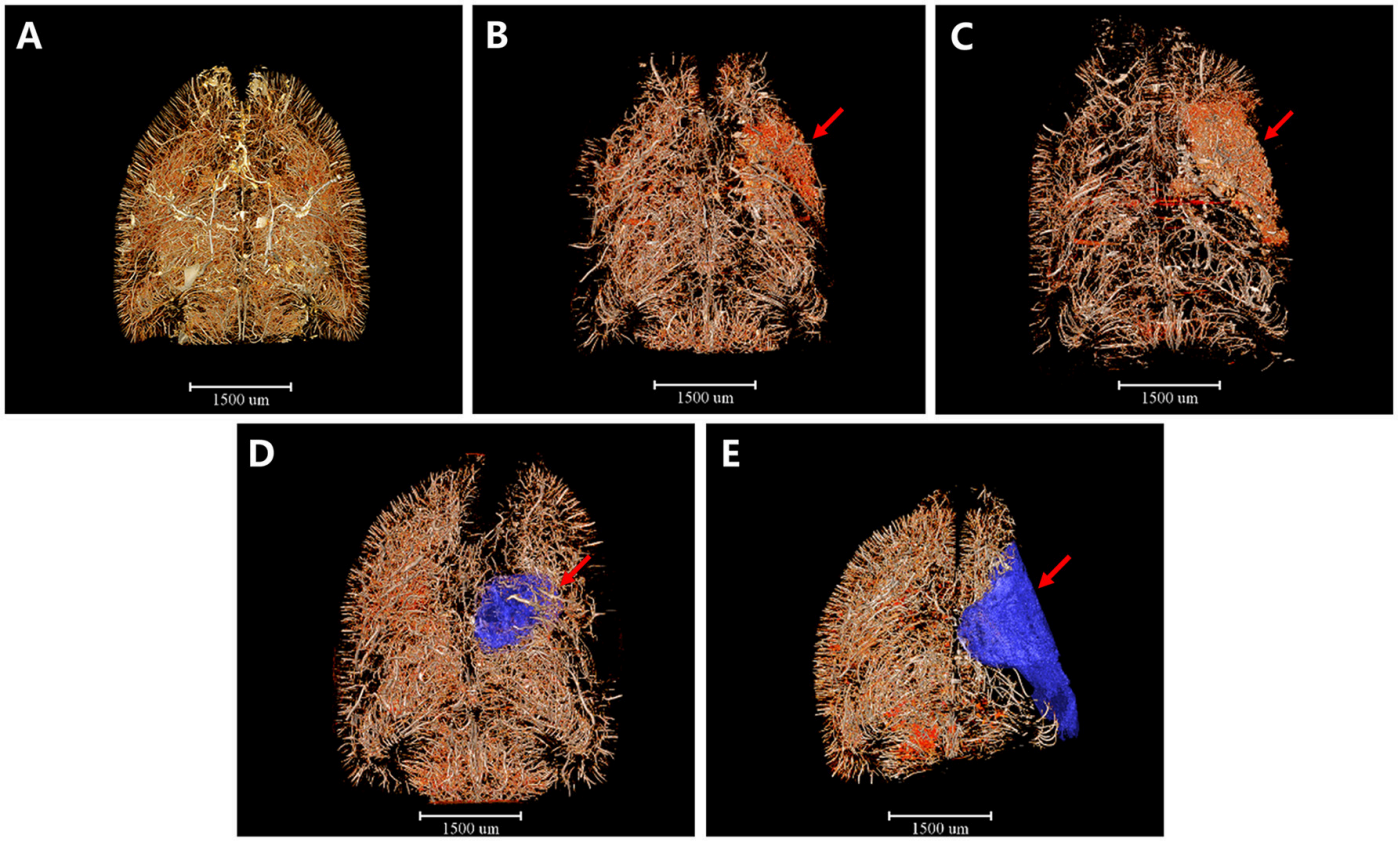
3D tomography of cerebral angioarchitecture following MCAO. Red region refers to region that microvessels accumulated and blue region presents ischemic cavity with no microvessels. **(A)** Sham operation group. **(B)** 4 h after MCAO. **(C)** 6 h after MCAO. Microvessels increased abruptly just 4 h after ischemia and kept increasing until 6 h post-ischemia (red arrow). **(D)** 3 d after MCAO. **(E)** 18 d after MCAO. Microvessels decreased dramatically and an ischemic cavity (blue region pointed by red arrow) formed 3d after MCAO. The ischemic cavity extended 18 d after MCAO. Scale bars: 1,500 μm.

Furthermore, we detected 3D architectural changes of the vascular network on the whole brain scale following ischemia injury with different time points and initially extracted integral focal cavity for the first time (Figure 2). We found that vessels around the focal lesion region increased at the 4th and 6th hour after ischemia, but were mostly short and discontinuous with disordered and distorted courses (Figures 2A–C). This might explain the expansion and opening of microvessels after MCAO. 3 days after ischemia, density of the focus decreased and cavity began to form (Figure 2D). Eighteen days after ischemia, a bigger cavity could be seen and the surrounding vascular network decreased markedly (Figure 2E). 3D maps with blue-cavity signal subtracted are shown in Supplementary Figure 1.

### 3D Angioarchitecture Maps Supplying the Pre-frontal Lobe Cortex and Corpus Striatum Region (Defined as the Region of Interest, ROI) After Ischemia in Coronary and Horizontal View

**Figure 3:**
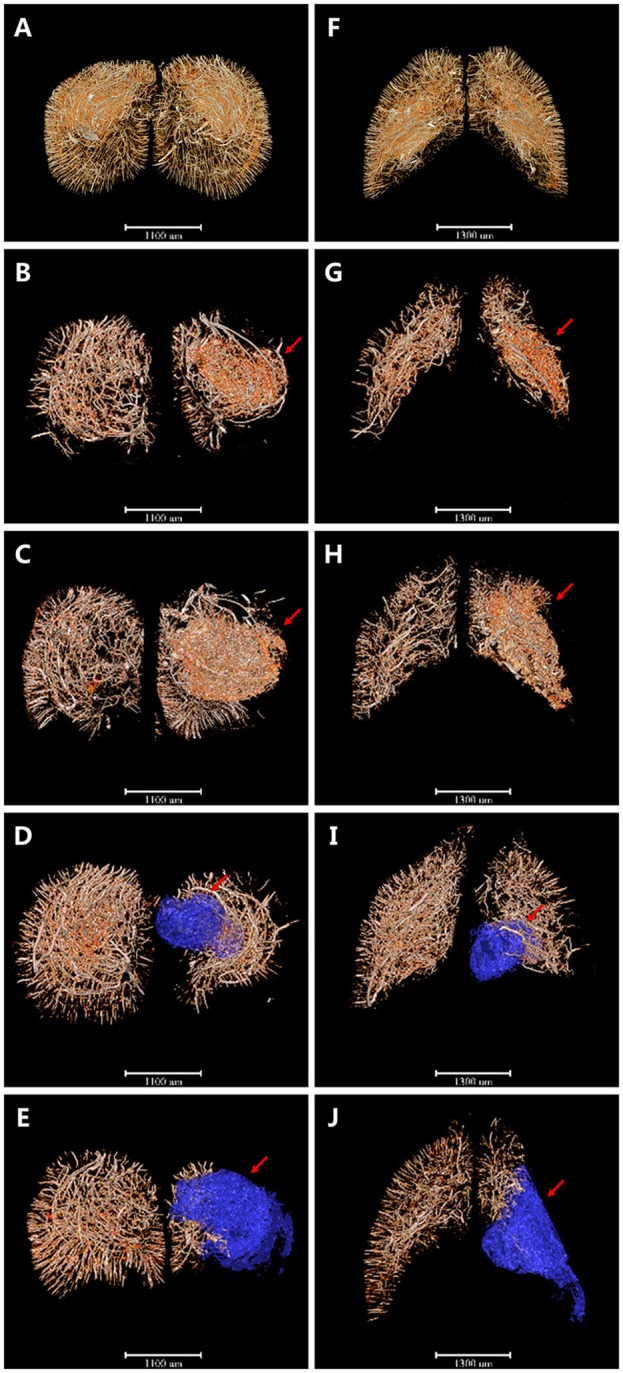
3D angioarchitecture maps supplying the pre-frontal lobe cortex and corpus striatum region (defined as the region of interest, ROI) after ischemia in coronary and horizontal view. Red region refers to region that microvessels accumulated and blue region presents ischemic cavity with no microvessels. **(A–E)** The coronary plane. **(F–J)** The horizontal plane. **(A,F)** sham operation group. **(B,G)** 4 h after MCAO. Microvessels with abundant branches began to appear around the ischemic area (red arrow). **(C,H)** 6 h after MCAO. Intensity of microvessels increased obviously but only seen surround the focus spatially (red arrow). **(D,I)** 3d after MCAO. Vasculature in ROI decreased significantly with liquefactive necrosis and ischemic cavity (blue region pointed by red arrow) formation. **(E,J)** 18 d after MCAO. Ischemic side of brain sagged and atrophied with vasculature attenuated dramatically and ischemic cavity (blue region pointed by red arrow) enlarged. Scale bars: 1,100 μm **(A–E)** and 1,300 μm **(F–J)**.

**Figure 4:**
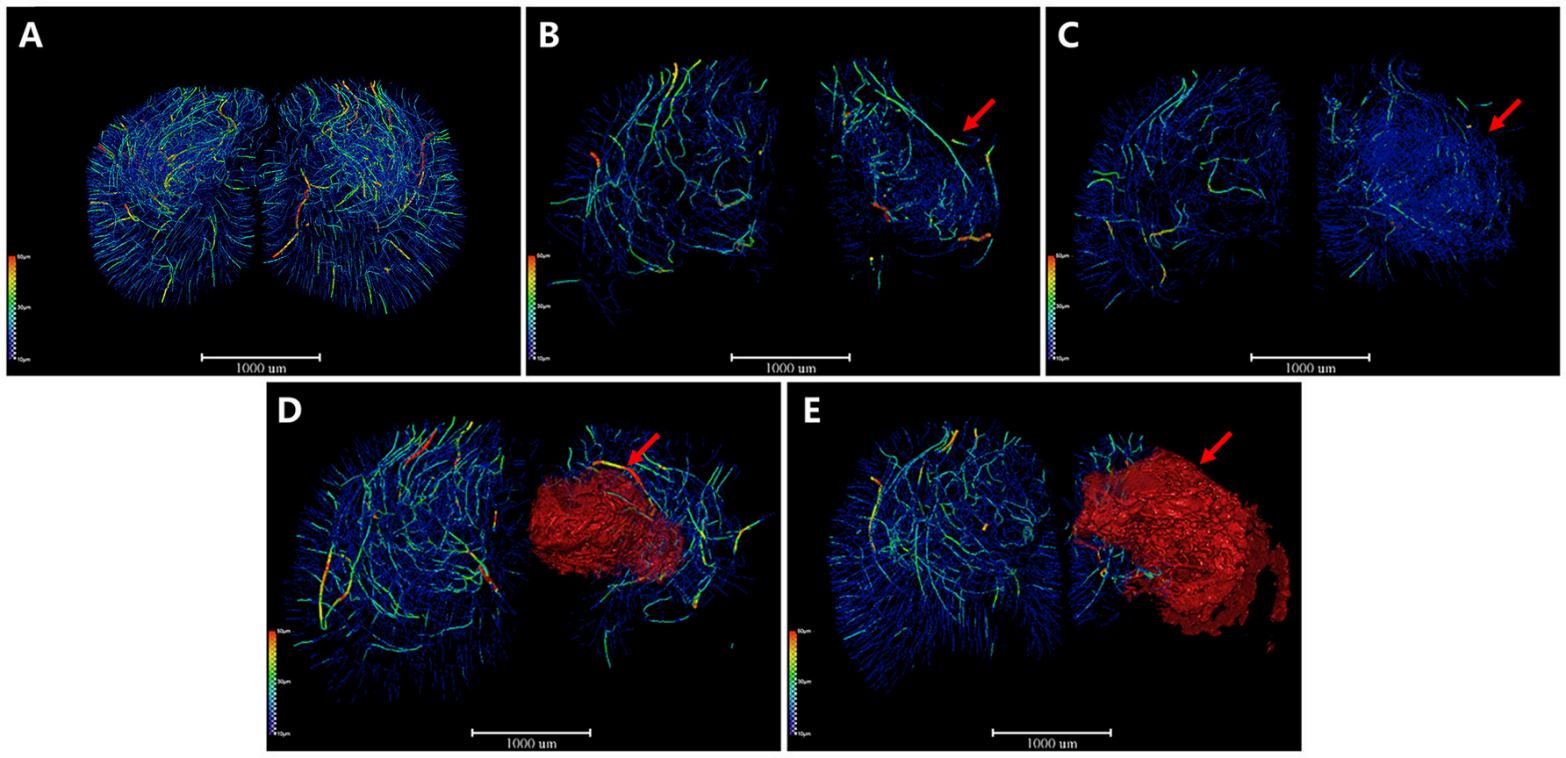
3D angioarchitectural keletonization of ROI after MCAO. The color gradients reflects vessel diameters ranging from 10 μm (dark blue) to 50 μm (red). Red mass (red arrow) refers to vessel-free cavity. **(A)** Sham operation group. 3D angioarchitectural keletonization of ROI was based on microvessels with diameters under 30 μm. **(B)** 4 h after MCAO. **(C)** 6 h after MCAO. Microvessels of diameters under 30 μm (blue vessels directed by red arrow) appeared around the ischemic region, representing newly open microvessels or angiogenesis. **(D)** 3 d after MCAO. **(E)** 18 d after MCAO. Newly appeared microvessels were gradually replaced by ischemic cavity (red mass pointed by red arrow). Scale bars: 1,000 μm.

Based on this, 3D angioarchitecture maps supplying the region of interest (ROI) after ischemia were extracted in both coronary and horizontal view. Here we defined the pre-frontal lobe cortex and corpus striatum region as the ROI after MCAO (Figure 3). Compared with the sham operation group (Figures 3A,F), 4 h after MCAO, irregular tube-like structures with abundant branches appeared densely around the ischemic area (red arrow). The newly-appeared microvessels, characterized by enhanced edges, were located around the dotted ischemic region with its number far more in the ischemic side than in the normal side (Figures 3B,G). Six hours after ischemia, the intensity of microvessels around the focus increased in an obvious manner. It is interesting that those blastogenetic microvessels did not appear in the center of the ischemic focus but were enriched surround it and grew in a manner that encircled the focus spatially (Figures 3C,H). As time went on, the integrity and organization of blood supply in the ischemic area were destructed progressively. Three days later, vasculature in the pre-frontal lobe cortex and corpus striatum decreased significantly, along with liquefactive necrosis and vessel-lack cavity formation (Figures 3D,I). Eighteen days later, the ischemic side of the brain sagged and atrophied with vasculature attenuated dramatically and the ischemic cavity enlarged (Figures 3E,J). All the above changes were revealed in detail through the cerebral vascular network reconstruction in multiple perspectives. 3D maps with blue-cavity signal subtracted are shown in Supplementary Figure 2.

### 3D Angioarchitectural Skeletonization of ROI After MCAO

Additionally, we extracted the corresponding 3D vascular network skeleton maps for a detailed analysis. Continuous pseudocolor alternations depicted the distribution range of the spectrum of vessel diameters (Figure 4). The color gradients represents vessel diameters, ranging from 10 μm (dark blue) to 50 μm (red). The red mass indicated the formation of focal cavity after MCAO. 3D angioarchitectural keletonization of ROI was based on microvessels with diameters under 30 μm (blue vessels in Figure 4A). The microvascular network began to reconstruct after ischemia. Specifically, at the 4th and 6th hour after ischemia, the destruction of vessels with a diameter >30 μm (green-yellow-red vessels) attenuated and vessels with a diameter of ~10 μm (dark blue) increased, forming dense a vascular network for compensation (Figures 4B,C). On the 3rd and 18th day after MCAO, especially in the latter case, a big cavity (red mass pointed by red arrow) was formed and the integrity of vasculature was destructed (Figures 4D,E). 3D maps with blue-cavity signal subtracted are shown in Supplementary Figure 3.

### CD31 Immunohistochemistry

**Figure 5:**
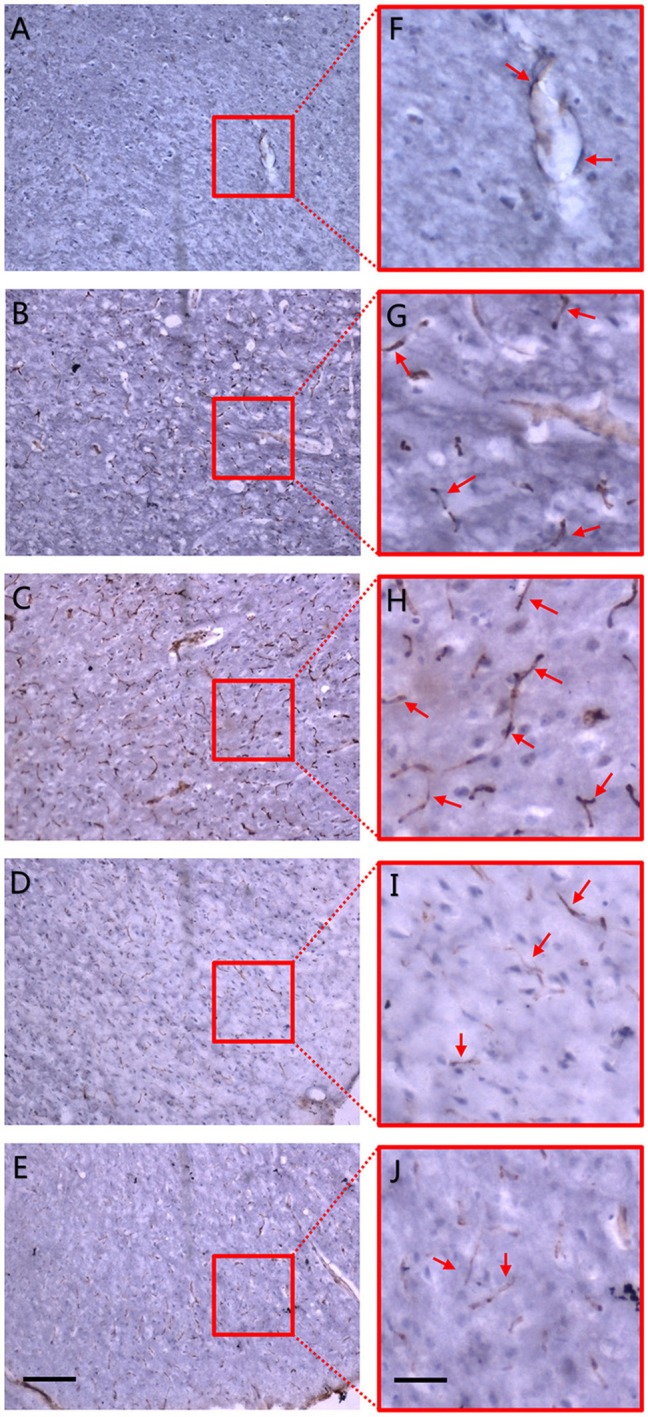
CD31 immunohistochemistry. Dotted and curved brown-staining particles represent membrane of vascular endothelium which were stained by CD31 antibody. Red arrows in right panel of magnified maps show positively-stained vascular endothelium. **(A)** Sham operation group. **(B)** 4 h after MCAO. **(C)** 6 h after MCAO. Dotted and curved brown-staining particles were seen as early as 4 h after MCAO and increased till 6 h after ischemia. **(D)** 3 d after MCAO. **(E)** 18 d after MCAO. Positive staining gradually faded away 3 d post-ischemia and only scattered vessels were remained on the 18th day after MCAO. Positive CD31 immunohistochemistry staining was defined as brown-staining particles located in the membrane of vascular endothelium. **(F–J)** Local magnification of figure **(A–E)**, respectively. Scale bars: 400 μm **(A–E)** and 100 μm **(F–J)**.

To further confirm the accuracy of synchrotron radiation imaging, we obtained CD31 immunohistochemistry pictures at the corpus striatum region (Figure 5). A positive result was defined as brown-staining particles located in the membrane of vascular endothelium (shown by red arrows in the right magnified panels). Compared with the sham operation group (Figure 5A), just 4 h after MCAO, positive staining could already be seen, mostly at the marginal area of the ischemic focus, depicting that dotted, curved, short rod-like, and circular tube-like microvessels ran in an irregular manner (Figure 5B). Six hours after ischemia, the number of small vessels raised in the focal edge while still having no regularity (Figure 5C). On the 3rd and 18th day, vasculature in the ischemic focus gradually faded away, leaving behind scattered vessels (Figures 5D,E). The tendency of microvessels described above was consistent with the results observed by synchrotron radiation imaging.

### 3D Quantitative Analysis

**Figure 6:**
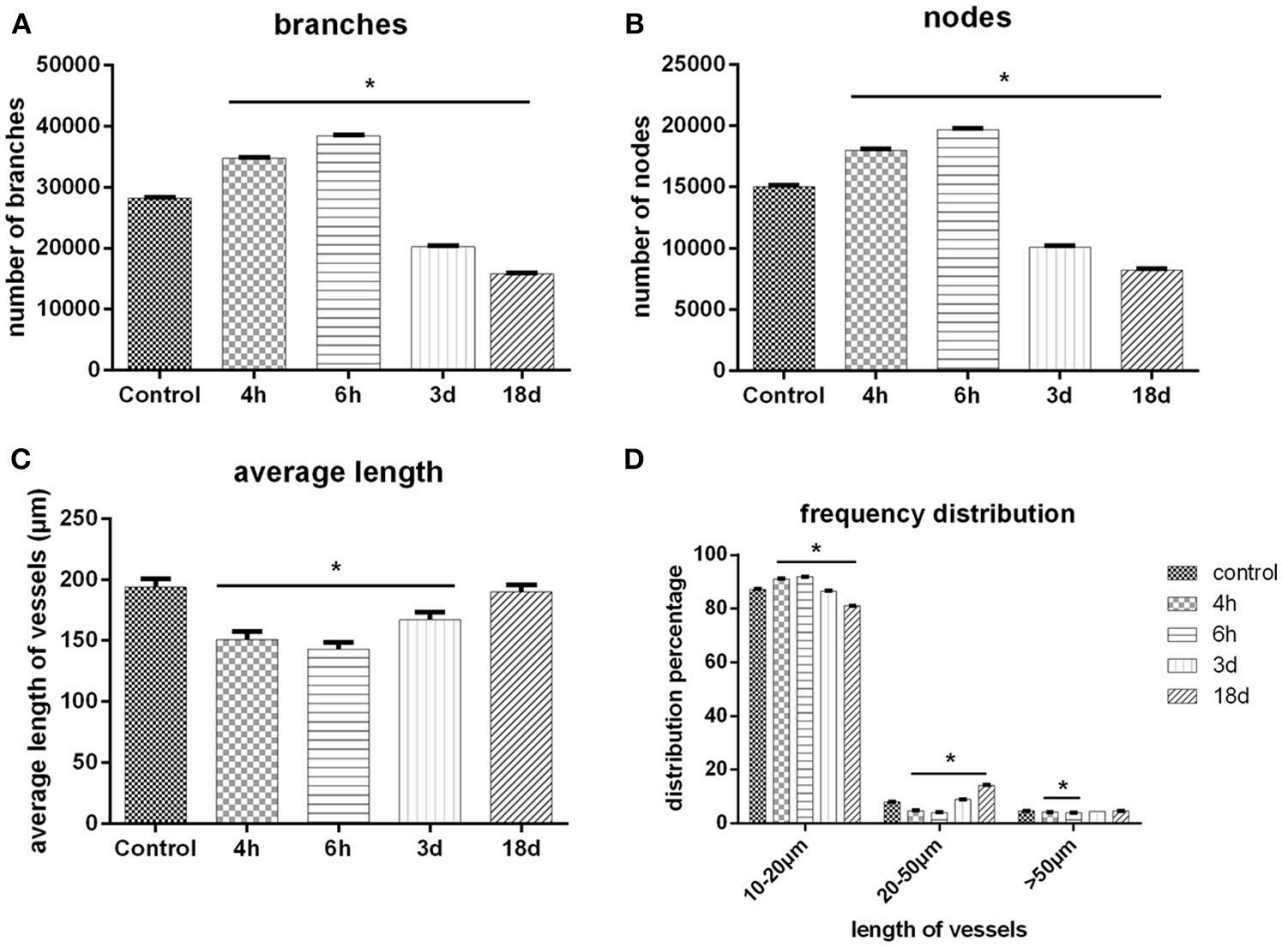
3D quantitative analysis. **(A)** Number changes of branches with time after MCAO. **(B)** Number changes of nodes with time after MCAO. **(C)** Length distribution of vessels at different time points after MCAO. **(D)** Quantity distribution of global vessels at different diameter ranges after MCAO. One-way ANOVA was used to analyze the difference between groups of each time point post-ischemia and the sham groups. ^*^p < 0.05 compared to the control group.

The important parameters of a cerebral angioarchitectural profile were deeply analyzed in Figure 6. The numbers of branches and nodes at 4th and 6th hour after ischemia were dramatically greater than in the sham operation group (*p* < 0.05), and as time went on, the numbers gradually decreased on the 3rd and 18th day after ischemia (*p* < 0.05) (Figures 6A,B). The average length of vessels at the 4th and 6th hour and on the 3rd day after ischemia were significantly decreased than in the sham operation group (*p* < 0.05), and as time went on, the average length of vessels was gradually raised (Figure 6C). We also analyzed the frequency distribution of the diameters of vessels, shown in Figure 6D. Data demonstrated that vessels with a diameter of under 20 μm were mostly responsible for the changes after MCAO. Four and Six hours after ischemia, the frequency of vessels with diameters of 10–20 μm were obviously increased and recorded a significant difference in comparison with the control group (*p* < 0.05). Over time, the frequency attenuated dramatically with significant difference on the 3rd and 18th day after ischemia (*p* < 0.05). Similar changes of frequency of vessels were shown in groups with diameters of 20–50 or over 50 μm.

## Discussion

More and more researches demonstrate that the microenvironmental changes after cerebral blood blockage could initiate cascade effects that induce spontaneous angiogenesis (Ergul et al., 2012; Yanev and Dijkhuizen, 2012), the extent of which may be crucial to the outcome of ischemic penumbra, the key point of clinical treatment of stroke. Clinical statistics showed that those stroke patients who had a higher density of new microvessels gained a better prognosis (Font et al., 2010). However, the point at which endogenous angiogenesis begins and how it develops remains unknown. To address these problems, it would be desirable if high-resolution vessel visualization of vasculature techniques were developed.

In the present study, we used SRμCT to rebuild the process of microvessel reconstruction in 3D prospective after ischemic stroke in rats and successfully extracted stroke cavity for microvessel visualization and quantitative analysis for the first time, trying to provide evidence for illustrating the pathogenesis of cerebrovascular diseases and for biological basis of targeted angiogenesis stimulator and inhibitor researches. 3D microvessel morphology can be visualized by reconstruction of the vascular skeleton, intuitionistically reflecting the real construction and its changes after ischemia. Besides, identifying vessels varying in diameters with distinguished colors greatly simplified the observation of complex microvascular distribution. Furthermore, a post-imaging process enables the description of the changes of branches, nodes, length, and diameter frequency of new vessels, quantitatively reflecting the vascular transformation after ischemia. To better identify the 3D luminal structure of the targeted vascular in detail, we utilized virtual micro-endoscopy for stereoscopic visualization of intravascular micro-structure and for the measurement of intravascular anatomy (Supplementary Video).

As early as 4 h after MCAO, we found that local microvasculature had already began to remodel. The increased microvessels were short, discontinuous, and highly twisty, arranged in a disordered pattern to form complex microvascular networks spatially surrounding the ischemic focus. We demonstrated that vascular density was still increasing 6 h after ischemia. At first, the new microvessels mostly localized at the ischemic penumbra around the injured area and then gradually stretched to the focal center but did not reach the focal center. As time went on, as was observed on the 3rd and 18th day, a cavity gradually formed in the center of corpus striatum because of a loss of blood supply, and the surrounding vascular density decreased. We have compared those views with a rat anatomical map (Scremin, 2004), making sure that the ROI area was consistent with the anatomical location of the rat map. A quantitative analysis of vasculature showed that the numbers of vascular branches and nodes at 4 h and 6 h after ischemia were significantly increased, in accordance with the fact that increased microvessels had abundant branches. 3 days later, the value began to decrease and a central cavity gradually formed but the growth of the surrounding micro-vessels still could be observed. 18 days after ischemia, the cavity enlarged and vascular density decreased dramatically. We assumed that the decrease may mainly come from 2 reasons. One was directly due to the decrease of microvessels and the other was that the formation of a cavity with no vessels in the late ischemic stage influenced the total measurement. Moreover, a vessel length analysis revealed that average vascular length of the whole brain reduced progressively from the 4–6th hour. This was because that new vessels were short and discontinuous, which influenced general measurement despite their increased density. 3 days later, the average vessel length began to rise and seemed to rise even more 18 days later, in accordance with the fact that newly generated vessels reduced and a cavity formed in the late stage of ischemia. To verify 3D visualization, we used immunohistochemistry to observe endogenous angiogenesis after ischemia. We found that the trend of vasculature in 2D histological slices were in consistent with the change of 3D general microvascular shape, thus providing evidence to the accuracy of SRμCT. Besides, by comparing 2D immunohistochemistry slides with SRμCT, we can see that the latter presents the huge advantage of showing 3D information without complex processing of samples.

Nevertheless, both 2D-and 3D-level evidences demonstrated that microvessels began to open and increase as early as 4 h after ischemia, trying to compensate the low transfusion, though these new vessels were mostly short and arranged in a disordered manner. Angiogenesis were mainly located around the edge of the ischemic area and gradually stretched to the center, indicating that the existing vasculature may be the structural basis of angiogenesis (Beck and Plate, 2009). However, this kind of compensation was transient and incomplete. In a later period, the vessels' density gradually decreased with glial cell proliferating, actively followed by cyst forming (Masamoto et al., 2012). As a result, tissues that were in the center of the focus necrosed because of ischemia, leaving a cavity in the center of the focus.

Ischemic stress activates a series of cascade responses which directly induce the necrosis and apoptosis of cerebral neurons, including disturbance of microcirculation, inflammatory responses, increased permeability of membranes to calcium ions, accumulation of neurotoxic amino acids, and free radical (Thored et al., 2007). Subsequently, the self-compensatory system initiates to establish three-level collateral circulation, in order to alleviate ischemic injury (Wittko-Schneider et al., 2014). To a certain extent, the compensation may not completely avoid the disruption of the cerebral vascular network, thus leading to neurological impairment. According to our research, the 3D visualization of cerebral vasculature showed that, as early as 4 h after MCAO of rats, local microvessels had already begun to open and increase, but as time went on, the vascular density decreased and finally ended up in cystic spaces, especially in the corpus striatum and the cortex region. Except for vascular density, diameter distribution frequency also changed. This was especially obvious in vessels with diameters of <20 μm. These changes have demonstrated decreased blood supply induced plasticity of local microvasculature at quite an early stage (Liu et al., 2018). As time went by, neurons in ischemic regions gradually died from continuous hypoxemia and were finally swallowed by inflammatory cells due to incomplete compensatory response (Manoonkitiwongsa et al., 2001; Liman and Endres, 2012; Masamoto et al., 2012).

Miao et al. (2016) investigated the changes of microvasculature in mouse brains following a 14-day reperfusion from transient MCAO by synchrotron radiation X-ray phase-contrast tomography (SRXPCT) with a pixel resolution of 3.7 × 3.7 μm, innovatively revealing active angiogenesis in the brain after stroke. In our present study, we performed permanent MCAO models in rats and succeeded to trace the process of vascular remodeling dynamically and spatially by directly extracting the ROI of the ischemic area with the SRμCT technique for the first time, offsetting the gap of early detection of general view of angiogenesis in the ischemic area after brain ischemia.

A great number of researches indicate that those with a higher density of neovasculature have both a higher survival rate and better outcomes (Zhang et al., 2000). So far, at least 3 potential mechanisms have been put forward to illustrate the stroke-protective effect of angiogenesis (Ergul et al., 2012). On the initiation and development of angiogenesis, most current researches mainly focus on the elevation of the angiogenesis-relating factor like VEGF(Marshall, 2004; Chu et al., 2012), and the routine method to testify the changes of focal vasculature is histological staining or vessel immunological staining, which, nevertheless, cannot present spatial structures of vasculature in the whole brain or ROI. Besides, information provided by these methods is usually lost for their 2D limitation and destructive sectioning process. For example, decreased vascular density in the histological section indicates that the number of vessels that pass through the section decreases. In a 3D spatial structure, nevertheless, there may be diverse situations that cause the changes in 2D slices, for instance, branches of small vessels have not formed; vascular orientation or curvature changes; basic structure of vascular network changes, and so on.

Compared with histological sections, the SRμCT technique, with its advantage of ultra-high resolution, provides images of capillaries on the micron scale. In addition, SRμCT does not need ultramicrotomy, thus avoiding destruction of the whole brain and loss of information. The current study enables us to randomly select the ROI area to track the progression of angiogenesis. Based on this, a 3D analysis of structural parameters of vascular network under pathological conditions was achieved qualitatively and quantitatively. In this study, we used both the SRμCT technique and the traditional immunohistochemical technique to show the dynamic process of microvascular reconstruction after ischemic injury in multiple perspectives. Because promoting functional angiogenesis may be important to improve self-rehabilitation, we may further apply the SRμCT technique as a powerful tool to evaluate the effectiveness of different therapies to positively regulate angiogenesis in a structure-function perspective.

Recently, light-sheet imaging has become a popular method due to improvements in imaging equipment (including microscopes and cameras), fluorescent probes, and image analysis techniques (de Medeiros et al., 2016; Strnad et al., 2016; Zundler et al., 2017). Being popular with researchers in neuroscience and developmental biology, light-sheet imaging can quickly obtain high-resolution 3D imaging of transparent samples with a relatively smaller volume. However, for large samples and those that are not so transparent, it is still a problem to figure out how to solve scattering and phase difference problems. In addition, attention needs to be paid to monitor the potential phototoxicity when conducting laser tomography. Aside from the fact that the specimens should be translucent by clarity technique before light-sheet microscope inspection, the preparation for SR-PCI scanning is not that complicated without any angiography, thus acquiring ultra-high resolution 3D images.

Due to its unique advantage of extremely high spatial resolution, using the SRμCT technique to explore clinical problems in a new prospective has been a new trend. It has been reported that SRμCT might reveal the truth in microvasculature changes in several common pathological processes like coronary embolism (Zhang et al., 2014a), hepatic cirrhosis (Fu et al., 2016), renal ischemia-reperfusion injury (Velroyen et al., 2014), spinal cord injury (Hu et al., 2015; Wu et al., 2018), inter-vertebral discs aging (Hu et al., 2017), and so on. SRμCT also shows its special superiority in researches on tumors such as lung metastatic cancers (Lin et al., 2015), breast tumors (Jian et al., 2015), and gastrointestinal cancers (Tang et al., 2012). However, the application of the SRμCT technique to identify angiogenesis immediately after cerebral ischemia has not been studied yet before us.

Although the SRμCT technique has a certain remarkable superiority over cerebrovasculature visualization, it still possesses some limitations. Since our present studies principally focus on establishing an imaging technique for *ex vivo* 3D quantitative analysis of the brain microvasculature, it is inevitable that the imaging process would be disturbed by blood flow, heartbeat, and respiration. Another issue is the simultaneous 3D imaging interaction of the micro-vascular network and the neuronal system. To our knowledge, Fratini et al. (2015) have already performed the 3D simultaneous visualization, in which the single neuron and vessel network were well-distinguishable in the spinal cord, confirming the possibility of simultaneous detection. However, their complex interaction has not been elucidated and there is still a large gap in the research field. In future studies, we will apply this higher resolution technique for the visualization of the interaction of a vessel structure and neural system. Additionally, since it requires long-time scanning, high-resolution imaging usually implies a high radiation dose, which doubtlessly produces radiation damage to the living specimen. Before the SRμCT technique is applied to clinical practice, how to enlarge imaging field, how to lower radiation damage, and how to optimize imaging conditions are unaddressed problems that require prompt solutions. With the improvement of the SRμCT technique, it will undoubtably play an important role in neural microcirculatory system studies.

## Conclusion

In this article, we depicted the dynamic changes of microvasculature in ROI in MACO rat model for the first time in both qualitative and quantitative respects using SRμCT. Vascular remodeling, including angiogenesis and the opening of microvessels, was found to start as early as 4 h after ischemia, which was, however, not so irregularly mapped, thus not so helpful to relieve ischemic stress of ischemic penumbra, indicating the great significance of early treatment to lead functional angiogenesis that saves ischemic penumbra as much as possible. Also, as an intuitionistic method to view microvasculature, SRμCT exhibits its unique superiority to explore the pathological process of microvessels and evaluate the effects of treatments of stroke.

## Author Contributions

MZ conceptualized the study, acquired funding, and administered the project. YL, SS, and XR curated the date, and wrote the original draft. XY worked on the methodology and software. MT provided the resources. YC supervised the study. XY, HZ, and ZW worked on validation. BX and MZ wrote, reviewed, and edited the manuscript.

### Conflict of Interest Statement

The authors declare that the research was conducted in the absence of any commercial or financial relationships that could be construed as a potential conflict of interest.

## Supplementary Material

Video S1 Virtual micro-endoscopy.
