# Supplementary material for: Non-destructive 3D Microtomography of Cerebral Angioarchitecture Changes Following Ischemic Stroke in Rats Using Synchrotron Radiation
Source: Front Neuroanat. 2019 Jan 31;13:5. doi: 10.3389/fnana.2019.00005 (PMC6365468; doi:10.3389/fnana.2019.00005)
Supplement: Supplementary file 1 [file Table_1.DOCX]

Supplementary Material

Nondestructive 3D microtomography of cerebral angioarchitecture changes following ischemic stroke in rats using synchrotron radiation

**Yonghong Luo ^1#^, Xianzhen Yin ^2#^, Shupeng Shi ^1^, Xiaolei Ren ^3^, Haoran Zhang ^1^, Zhuolu Wang ^4,5^, Yong Cao ^6^, Mimi Tang ^7,8^, Bo Xiao ^1^, Mengqi Zhang ^1*^**

*** Correspondence:** Mengqi Zhang: zhangmengqi8912@163.com

# Supplementary Data

**1.1 Raw data**

**Table 1. Number of branches in varied time points after MCAO in rats.**

|  | control | 4h | 6h | 3d | 18d |
| --- | --- | --- | --- | --- | --- |
| 1 | 28368 | 34920 | 38561 | 20376 | 15935 |
| 2 | 28107 | 34647 | 38297 | 20098 | 15635 |
| 3 | 28257 | 34758 | 38438 | 20249 | 15797 |
| mean±SD | 28244±131 | 34775±137 | 38432±132 | 20241±139 | 15789±150 |

**Table 2. Number of nodes in varied time points after MCAO in rats.**

|  | control | 4h | 6h | 3d | 18d |
| --- | --- | --- | --- | --- | --- |
| 1 | 15170 | 18114 | 19789 | 10220 | 8346 |
| 2 | 14932 | 17878 | 19529 | 10012 | 8125 |
| 3 | 15057 | 18005 | 19680 | 10134 | 8255 |
| mean±SD | 15053±119 | 17999±118 | 19666±131 | 10122±105 | 8242±111 |

**Table 3. Average length of vessels (μm) in varied time points after MCAO in rats.**

|  | control | 4h | 6h | 3d | 18d |
| --- | --- | --- | --- | --- | --- |
| 1 | 200.8 | 156.7 | 148.2 | 173.3 | 195.2 |
| 2 | 186.7 | 143.4 | 136.1 | 160.0 | 184.3 |
| 3 | 194.2 | 152.0 | 144.1 | 167.7 | 190.8 |
| mean±SD | 193.9±7.1 | 150.7±6.7 | 142.8±6.2 | 167±6.7 | 190.1±5.5 |

**Table 4. Distribution frequency of vessels in varied time points after MCAO in rats.**

| 10-20μm |  | control | 4h | 6h | 3d | 18d |
| --- | --- | --- | --- | --- | --- | --- |
|  | 1 | 87.39 | 91.21 | 92.02 | 86.72 | 81.19 |
|  | 2 | 87.24 | 90.99 | 91.86 | 86.56 | 81.06 |
|  | 3 | 87.30 | 91.16 | 91.97 | 86.67 | 81.14 |
|  | mean±SD | 87.31±0.08 | 91.12±0.12 | 91.95±0.08 | 86.65±0.08 | 81.13±0.07 |
| 20-50μm |  | control | 4h | 6h | 3d | 18d |
|  | 1 | 8.07 | 4.62 | 3.98 | 8.79 | 14.13 |
|  | 2 | 8.15 | 4.75 | 4.09 | 8.96 | 14.33 |
|  | 3 | 8.08 | 4.7 | 4.02 | 8.86 | 14.26 |
|  | mean±SD | 8.1±0.04 | 4.69±0.07 | 4.03±0.06 | 8.87±0.09 | 14.24±0.1 |
| >50μm |  | control | 4h | 6h | 3d | 18d |
|  | 1 | 4.54 | 4.17 | 4.00 | 4.49 | 4.68 |
|  | 2 | 4.61 | 4.26 | 4.05 | 4.48 | 4.61 |
|  | 3 | 4.62 | 4.14 | 4.01 | 4.47 | 4.60 |
|  | mean±SD | 4.59±0.04 | 4.19±0.06 | 4.02±0.03 | 4.48±0.01 | 4.63±0.04 |

**1.2 Data analysis**

Data analysis was performed with SPSS 17.0 (SPSS, Inc., Chicago, IL, USA). The data in each group is normally distributed tested by Shapiro-Wilk test with a p-value >0.05. Test of Homogeneity shows no heterogeneity in data between groups with p-values >0.05. The data were analyzed with one-way analysis of variance (ANOVA) followed by Tukey’s method for the post hoc test to observe the effect of time post-ischemia on the morphological parameters. p-value<0.05 was considered to be significant.

**Test of Shapiro-Wilk for Gaussian distribution**

|  | time | Shapiro-Wilk | | |
| --- | --- | --- | --- | --- |
|  |  | Statistic | df | Sig. |
| branches | control | .993 | 3 | .836 |
|  | 4h | .989 | 3 | .795 |
|  | 6h | .998 | 3 | .925 |
|  | 3d | .998 | 3 | .905 |
|  | 18d | .998 | 3 | .912 |
| nodes | control | .999 | 3 | .944 |
|  | 4h | .998 | 3 | .916 |
|  | 6h | .991 | 3 | .822 |
|  | 3d | .990 | 3 | .810 |
|  | 18d | .990 | 3 | .806 |
| average_length | control | .999 | 3 | .930 |
|  | 4h | .972 | 3 | .680 |
|  | 6h | .967 | 3 | .649 |
|  | 3d | .992 | 3 | .826 |
|  | 18d | .988 | 3 | .788 |
| 10-20μm | control | .987 | 3 | .780 |
|  | 4h | .910 | 3 | .417 |
|  | 6h | .955 | 3 | .593 |
|  | 3d | .955 | 3 | .593 |
|  | 18d | .983 | 3 | .747 |
| 20-50μm | control | .842 | 3 | .220 |
|  | 4h | .983 | 3 | .747 |
|  | 6h | .976 | 3 | .702 |
|  | 3d | .990 | 3 | .806 |
|  | 18d | .971 | 3 | .672 |
| >50μm | control | .842 | 3 | .220 |
|  | 4h | .923 | 3 | .463 |
|  | 6h | .893 | 3 | .363 |
|  | 3d | 1.000 | 3 | 1.000 |
|  | 18d | .842 | 3 | .220 |

| **Test of Homogeneity of Variances** | | | | |
| --- | --- | --- | --- | --- |
|  | Levene Statistic | df1 | df2 | Sig. |
| branches | .014 | 4 | 10 | 1.000 |
| nodes | .034 | 4 | 10 | .997 |
| average_length | .046 | 4 | 10 | .995 |
| 10-20μm | .488 | 4 | 10 | .745 |
| 20-50μm | .634 | 4 | 10 | .650 |
| >50μm | 2.811 | 4 | 10 | .084 |

| **ANOVA** | | | | | |
| --- | --- | --- | --- | --- | --- |
|  | | Sum of Squares | df | Mean Square | F |
| branches | Between Groups | 1088484944.400 | 4 | 272121236.100 | 14266.007 |
|  | Within Groups | 190748.000 | 10 | 19074.800 |  |
|  | Total | 1088675692.400 | 14 |  |  |
| nodes | Between Groups | 291491007.600 | 4 | 72872751.900 | 5324.465 |
|  | Within Groups | 136864.000 | 10 | 13686.400 |  |
|  | Total | 291627871.600 | 14 |  |  |
| average_length | Between Groups | 6271.500 | 4 | 1567.875 | 37.727 |
|  | Within Groups | 415.580 | 10 | 41.558 |  |
|  | Total | 6687.080 | 14 |  |  |
| 10-20μm | Between Groups | 222.466 | 4 | 55.616 | 7577.174 |
|  | Within Groups | .073 | 10 | .007 |  |
|  | Total | 222.539 | 14 |  |  |
| 20-50μm | Between Groups | 199.262 | 4 | 49.815 | 9259.366 |
|  | Within Groups | .054 | 10 | .005 |  |
|  | Total | 199.315 | 14 |  |  |
| >50μm | Between Groups | .847 | 4 | .212 | 124.535 |
|  | Within Groups | .017 | 10 | .002 |  |
|  | Total | .864 | 14 |  |  |

| **ANOVA** | | |
| --- | --- | --- |
|  | | Sig. |
| branches | Between Groups | .000 |
|  | Within Groups |  |
|  | Total |  |
| nodes | Between Groups | .000 |
|  | Within Groups |  |
|  | Total |  |
| average_length | Between Groups | .000 |
|  | Within Groups |  |
|  | Total |  |
| 10-20μm | Between Groups | .000 |
|  | Within Groups |  |
|  | Total |  |
| 20-50μm | Between Groups | .000 |
|  | Within Groups |  |
|  | Total |  |
| >50μm | Between Groups | .000 |
|  | Within Groups |  |
|  | Total |  |

**Post Hoc Tests**

| **Multiple Comparisons** | | | | | | | |
| --- | --- | --- | --- | --- | --- | --- | --- |
| Tukey HSD | | | | | | | |
| Dependent Variable | (I) time | (J) time | Mean Difference (I-J) | Std. Error | Sig. | 95% Confidence Interval | |
|  |  |  |  |  |  | Lower Bound | Upper Bound |
| branches | control | 4h | -6531.00000^*^ | 112.76761 | .000 | -6902.1275 | -6159.8725 |
|  |  | 6h | -10188.00000^*^ | 112.76761 | .000 | -10559.1275 | -9816.8725 |
|  |  | 3d | 8003.00000^*^ | 112.76761 | .000 | 7631.8725 | 8374.1275 |
|  |  | 18d | 12455.00000^*^ | 112.76761 | .000 | 12083.8725 | 12826.1275 |
|  | 4h | control | 6531.00000^*^ | 112.76761 | .000 | 6159.8725 | 6902.1275 |
|  |  | 6h | -3657.00000^*^ | 112.76761 | .000 | -4028.1275 | -3285.8725 |
|  |  | 3d | 14534.00000^*^ | 112.76761 | .000 | 14162.8725 | 14905.1275 |
|  |  | 18d | 18986.00000^*^ | 112.76761 | .000 | 18614.8725 | 19357.1275 |
|  | 6h | control | 10188.00000^*^ | 112.76761 | .000 | 9816.8725 | 10559.1275 |
|  |  | 4h | 3657.00000^*^ | 112.76761 | .000 | 3285.8725 | 4028.1275 |
|  |  | 3d | 18191.00000^*^ | 112.76761 | .000 | 17819.8725 | 18562.1275 |
|  |  | 18d | 22643.00000^*^ | 112.76761 | .000 | 22271.8725 | 23014.1275 |
|  | 3d | control | -8003.00000^*^ | 112.76761 | .000 | -8374.1275 | -7631.8725 |
|  |  | 4h | -14534.00000^*^ | 112.76761 | .000 | -14905.1275 | -14162.8725 |
|  |  | 6h | -18191.00000^*^ | 112.76761 | .000 | -18562.1275 | -17819.8725 |
|  |  | 18d | 4452.00000^*^ | 112.76761 | .000 | 4080.8725 | 4823.1275 |
|  | 18d | control | -12455.00000^*^ | 112.76761 | .000 | -12826.1275 | -12083.8725 |
|  |  | 4h | -18986.00000^*^ | 112.76761 | .000 | -19357.1275 | -18614.8725 |
|  |  | 6h | -22643.00000^*^ | 112.76761 | .000 | -23014.1275 | -22271.8725 |
|  |  | 3d | -4452.00000^*^ | 112.76761 | .000 | -4823.1275 | -4080.8725 |
| nodes | control | 4h | -2946.00000^*^ | 95.52103 | .000 | -3260.3675 | -2631.6325 |
|  |  | 6h | -4613.00000^*^ | 95.52103 | .000 | -4927.3675 | -4298.6325 |
|  |  | 3d | 4931.00000^*^ | 95.52103 | .000 | 4616.6325 | 5245.3675 |
|  |  | 18d | 6811.00000^*^ | 95.52103 | .000 | 6496.6325 | 7125.3675 |
|  | 4h | control | 2946.00000^*^ | 95.52103 | .000 | 2631.6325 | 3260.3675 |
|  |  | 6h | -1667.00000^*^ | 95.52103 | .000 | -1981.3675 | -1352.6325 |
|  |  | 3d | 7877.00000^*^ | 95.52103 | .000 | 7562.6325 | 8191.3675 |
|  |  | 18d | 9757.00000^*^ | 95.52103 | .000 | 9442.6325 | 10071.3675 |
|  | 6h | control | 4613.00000^*^ | 95.52103 | .000 | 4298.6325 | 4927.3675 |
|  |  | 4h | 1667.00000^*^ | 95.52103 | .000 | 1352.6325 | 1981.3675 |
|  |  | 3d | 9544.00000^*^ | 95.52103 | .000 | 9229.6325 | 9858.3675 |
|  |  | 18d | 11424.00000^*^ | 95.52103 | .000 | 11109.6325 | 11738.3675 |
|  | 3d | control | -4931.00000^*^ | 95.52103 | .000 | -5245.3675 | -4616.6325 |
|  |  | 4h | -7877.00000^*^ | 95.52103 | .000 | -8191.3675 | -7562.6325 |
|  |  | 6h | -9544.00000^*^ | 95.52103 | .000 | -9858.3675 | -9229.6325 |
|  |  | 18d | 1880.00000^*^ | 95.52103 | .000 | 1565.6325 | 2194.3675 |
|  | 18d | control | -6811.00000^*^ | 95.52103 | .000 | -7125.3675 | -6496.6325 |
|  |  | 4h | -9757.00000^*^ | 95.52103 | .000 | -10071.3675 | -9442.6325 |
|  |  | 6h | -11424.00000^*^ | 95.52103 | .000 | -11738.3675 | -11109.6325 |
|  |  | 3d | -1880.00000^*^ | 95.52103 | .000 | -2194.3675 | -1565.6325 |
| average_length | control | 4h | 43.20000^*^ | 5.26359 | .000 | 25.8771 | 60.5229 |
|  |  | 6h | 51.10000^*^ | 5.26359 | .000 | 33.7771 | 68.4229 |
|  |  | 3d | 26.90000^*^ | 5.26359 | .003 | 9.5771 | 44.2229 |
|  |  | 18d | 3.80000 | 5.26359 | .947 | -13.5229 | 21.1229 |
|  | 4h | control | -43.20000^*^ | 5.26359 | .000 | -60.5229 | -25.8771 |
|  |  | 6h | 7.90000 | 5.26359 | .584 | -9.4229 | 25.2229 |
|  |  | 3d | -16.30000 | 5.26359 | .068 | -33.6229 | 1.0229 |
|  |  | 18d | -39.40000^*^ | 5.26359 | .000 | -56.7229 | -22.0771 |
|  | 6h | control | -51.10000^*^ | 5.26359 | .000 | -68.4229 | -33.7771 |
|  |  | 4h | -7.90000 | 5.26359 | .584 | -25.2229 | 9.4229 |
|  |  | 3d | -24.20000^*^ | 5.26359 | .007 | -41.5229 | -6.8771 |
|  |  | 18d | -47.30000^*^ | 5.26359 | .000 | -64.6229 | -29.9771 |
|  | 3d | control | -26.90000^*^ | 5.26359 | .003 | -44.2229 | -9.5771 |
|  |  | 4h | 16.30000 | 5.26359 | .068 | -1.0229 | 33.6229 |
|  |  | 6h | 24.20000^*^ | 5.26359 | .007 | 6.8771 | 41.5229 |
|  |  | 18d | -23.10000^*^ | 5.26359 | .009 | -40.4229 | -5.7771 |
|  | 18d | control | -3.80000 | 5.26359 | .947 | -21.1229 | 13.5229 |
|  |  | 4h | 39.40000^*^ | 5.26359 | .000 | 22.0771 | 56.7229 |
|  |  | 6h | 47.30000^*^ | 5.26359 | .000 | 29.9771 | 64.6229 |
|  |  | 3d | 23.10000^*^ | 5.26359 | .009 | 5.7771 | 40.4229 |
| 10-20μm | control | 4h | -3.81000^*^ | .06995 | .000 | -4.0402 | -3.5798 |
|  |  | 6h | -4.64000^*^ | .06995 | .000 | -4.8702 | -4.4098 |
|  |  | 3d | .66000^*^ | .06995 | .000 | .4298 | .8902 |
|  |  | 18d | 6.18000^*^ | .06995 | .000 | 5.9498 | 6.4102 |
|  | 4h | control | 3.81000^*^ | .06995 | .000 | 3.5798 | 4.0402 |
|  |  | 6h | -.83000^*^ | .06995 | .000 | -1.0602 | -.5998 |
|  |  | 3d | 4.47000^*^ | .06995 | .000 | 4.2398 | 4.7002 |
|  |  | 18d | 9.99000^*^ | .06995 | .000 | 9.7598 | 10.2202 |
|  | 6h | control | 4.64000^*^ | .06995 | .000 | 4.4098 | 4.8702 |
|  |  | 4h | .83000^*^ | .06995 | .000 | .5998 | 1.0602 |
|  |  | 3d | 5.30000^*^ | .06995 | .000 | 5.0698 | 5.5302 |
|  |  | 18d | 10.82000^*^ | .06995 | .000 | 10.5898 | 11.0502 |
|  | 3d | control | -.66000^*^ | .06995 | .000 | -.8902 | -.4298 |
|  |  | 4h | -4.47000^*^ | .06995 | .000 | -4.7002 | -4.2398 |
|  |  | 6h | -5.30000^*^ | .06995 | .000 | -5.5302 | -5.0698 |
|  |  | 18d | 5.52000^*^ | .06995 | .000 | 5.2898 | 5.7502 |
|  | 18d | control | -6.18000^*^ | .06995 | .000 | -6.4102 | -5.9498 |
|  |  | 4h | -9.99000^*^ | .06995 | .000 | -10.2202 | -9.7598 |
|  |  | 6h | -10.82000^*^ | .06995 | .000 | -11.0502 | -10.5898 |
|  |  | 3d | -5.52000^*^ | .06995 | .000 | -5.7502 | -5.2898 |
| 20-50μm | control | 4h | 3.41000^*^ | .05989 | .000 | 3.2129 | 3.6071 |
|  |  | 6h | 4.07000^*^ | .05989 | .000 | 3.8729 | 4.2671 |
|  |  | 3d | -.77000^*^ | .05989 | .000 | -.9671 | -.5729 |
|  |  | 18d | -6.14000^*^ | .05989 | .000 | -6.3371 | -5.9429 |
|  | 4h | control | -3.41000^*^ | .05989 | .000 | -3.6071 | -3.2129 |
|  |  | 6h | .66000^*^ | .05989 | .000 | .4629 | .8571 |
|  |  | 3d | -4.18000^*^ | .05989 | .000 | -4.3771 | -3.9829 |
|  |  | 18d | -9.55000^*^ | .05989 | .000 | -9.7471 | -9.3529 |
|  | 6h | control | -4.07000^*^ | .05989 | .000 | -4.2671 | -3.8729 |
|  |  | 4h | -.66000^*^ | .05989 | .000 | -.8571 | -.4629 |
|  |  | 3d | -4.84000^*^ | .05989 | .000 | -5.0371 | -4.6429 |
|  |  | 18d | -10.21000^*^ | .05989 | .000 | -10.4071 | -10.0129 |
|  | 3d | control | .77000^*^ | .05989 | .000 | .5729 | .9671 |
|  |  | 4h | 4.18000^*^ | .05989 | .000 | 3.9829 | 4.3771 |
|  |  | 6h | 4.84000^*^ | .05989 | .000 | 4.6429 | 5.0371 |
|  |  | 18d | -5.37000^*^ | .05989 | .000 | -5.5671 | -5.1729 |
|  | 18d | control | 6.14000^*^ | .05989 | .000 | 5.9429 | 6.3371 |
|  |  | 4h | 9.55000^*^ | .05989 | .000 | 9.3529 | 9.7471 |
|  |  | 6h | 10.21000^*^ | .05989 | .000 | 10.0129 | 10.4071 |
|  |  | 3d | 5.37000^*^ | .05989 | .000 | 5.1729 | 5.5671 |
| >50μm | control | 4h | .40000^*^ | .03367 | .000 | .2892 | .5108 |
|  |  | 6h | .57000^*^ | .03367 | .000 | .4592 | .6808 |
|  |  | 3d | .11000 | .03367 | .052 | -.0008 | .2208 |
|  |  | 18d | -.04000 | .03367 | .758 | -.1508 | .0708 |
|  | 4h | control | -.40000^*^ | .03367 | .000 | -.5108 | -.2892 |
|  |  | 6h | .17000^*^ | .03367 | .004 | .0592 | .2808 |
|  |  | 3d | -.29000^*^ | .03367 | .000 | -.4008 | -.1792 |
|  |  | 18d | -.44000^*^ | .03367 | .000 | -.5508 | -.3292 |
|  | 6h | control | -.57000^*^ | .03367 | .000 | -.6808 | -.4592 |
|  |  | 4h | -.17000^*^ | .03367 | .004 | -.2808 | -.0592 |
|  |  | 3d | -.46000^*^ | .03367 | .000 | -.5708 | -.3492 |
|  |  | 18d | -.61000^*^ | .03367 | .000 | -.7208 | -.4992 |
|  | 3d | control | -.11000 | .03367 | .052 | -.2208 | .0008 |
|  |  | 4h | .29000^*^ | .03367 | .000 | .1792 | .4008 |
|  |  | 6h | .46000^*^ | .03367 | .000 | .3492 | .5708 |
|  |  | 18d | -.15000^*^ | .03367 | .008 | -.2608 | -.0392 |
|  | 18d | control | .04000 | .03367 | .758 | -.0708 | .1508 |
|  |  | 4h | .44000^*^ | .03367 | .000 | .3292 | .5508 |
|  |  | 6h | .61000^*^ | .03367 | .000 | .4992 | .7208 |
|  |  | 3d | .15000^*^ | .03367 | .008 | .0392 | .2608 |

| *. The mean difference is significant at the 0.05 level. |
| --- |

# Supplementary Figures

## Supplementary Figure 1

**
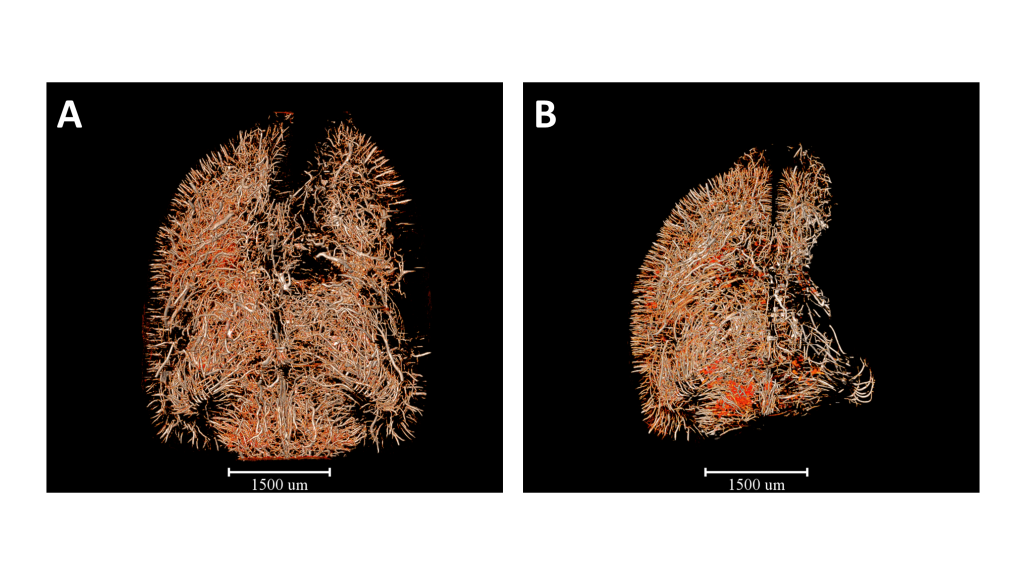
**

**Supplementary figure 1. 3D tomography of cerebral angioarchitecture 3d/18d after MCAO with the blue-cavity signal subtracted. (A)** 3d after MCAO (corresponding to figure 2**D**). Microvessels decreased dramatically and an ischemic cavity formed 3d after MCAO. The ischemic cavity extended 18d after MCAO. **(B)** 18d after MCAO (corresponding to figure 2**E**). Scale bars: 1500μm.

## Supplementary Figure 2

**
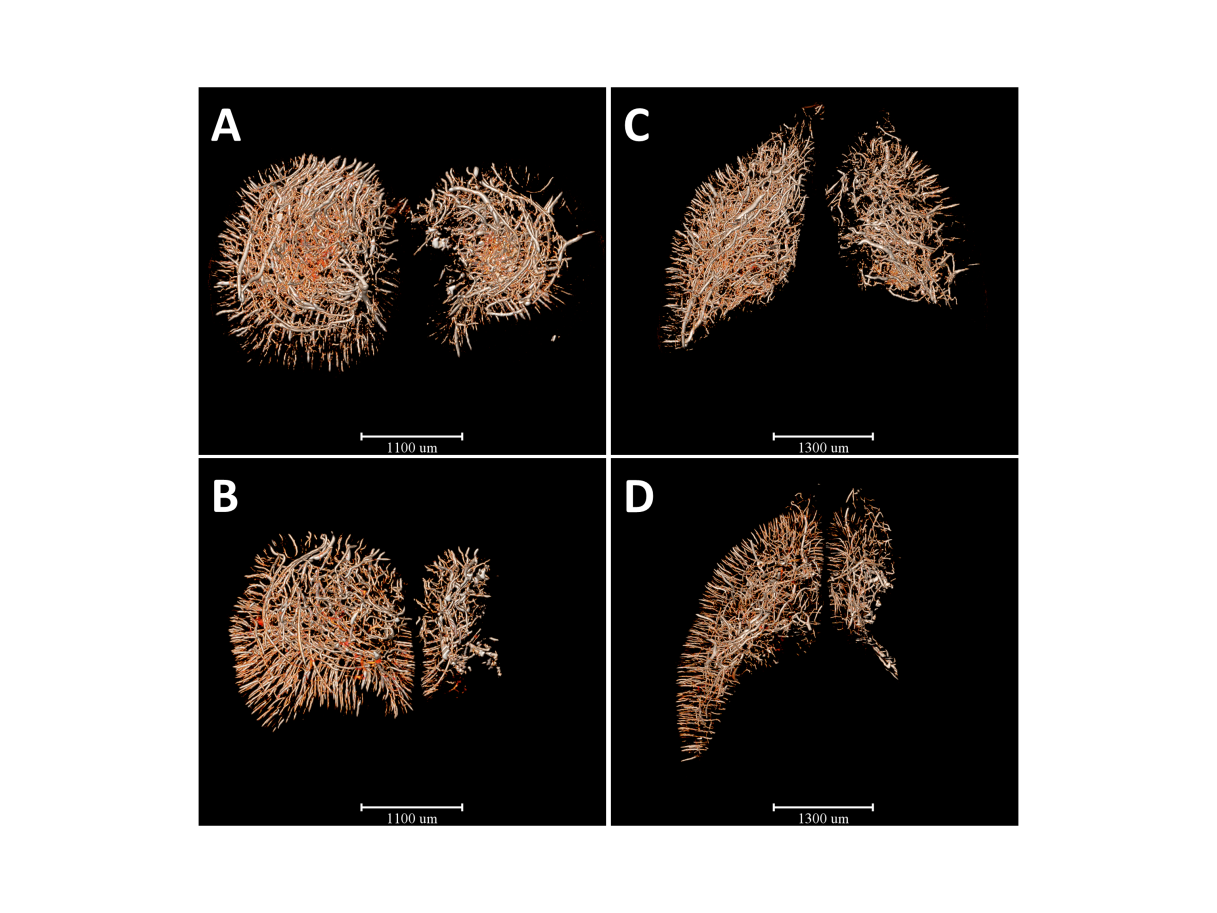
**

**Supplementary figure 2. 3D angioarchitecture maps with the blue-cavity signal subtracted supplying the pre-frontal lobe cortex and corpus striatum region (defined as the region of interest, ROI) after ischemia in coronary and horizontal view. (A)-(B)** The coronary plane. **(C)-(D)** The horizontal plane. (**A, C**) 3d after MCAO (corresponding to figure 3**D** and 3**I**, respectively). (**B, D**) 18d after MCAO (corresponding to figure 3**E** and 3**J**, respectively). Ischemic side of brain sagged and atrophied with vasculature attenuated dramatically and ischemic cavity formed and enlarged. Scale bars: 1100μm (**A, B**) and 1300μm (**C, D**).

## Supplementary Figure 3

**
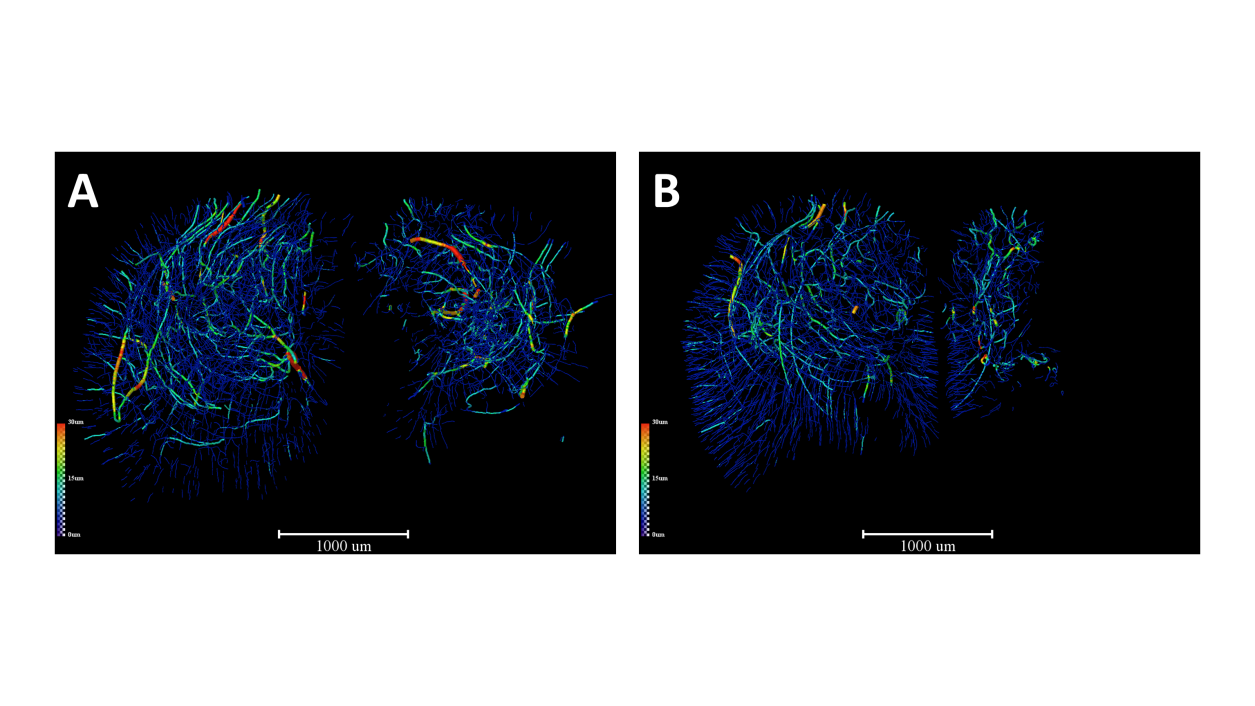
**

**Supplementary figure 3. 3D angioarchitectural keletonization of ROI with the blue-cavity signal subtracted after MCAO.** The color gradients reflects vessel diameters ranging from 10μm (dark blue) to 50μm (red). **(A)** 3d after MCAO (corresponding to figure 4**D**). **(B)** 18d after MCAO (corresponding to figure 4**E**). Ischemic cavity gradually formed in the ROI. Scale bars: 1000μm.

# Supplementary video.
